# Dementia in Fragile X-associated Tremor/Ataxia Syndrome

**DOI:** 10.1590/S1980-57642010DN40100014

**Published:** 2010

**Authors:** Ricardo Nitrini, Márcia Rúbia R. Gonçalves, Leonardo P. Capelli, Egberto Reis Barbosa, Cláudia Sellitto Porto, Edson Amaro, Paulo Alberto Otto, Angela M. Vianna-Morgante

**Affiliations:** 1 MD, Departments of Neurology, School of Medicine, University of São Paulo, São Paulo SP, Brazil.; 2 MSc, Department of Genetics and Evolutionary Biology, Institute of Biosciences, University of São Paulo, São Paulo SP, Brazil.; 3 PhD, Departments of Neurology, School of Medicine, University of São Paulo, São Paulo SP, Brazil.; 4 MD, Departments of Neurology and Radiology, School of Medicine, University of São Paulo, São Paulo SP, Brazil.; 5 MD, Department of Genetics and Evolutionary Biology, Institute of Biosciences, University of São Paulo, São Paulo SP, Brazil.; 6 PhD, Department of Genetics and Evolutionary Biology, Institute of Biosciences, University of São Paulo, São Paulo SP, Brazil.

**Keywords:** fragile X, dementia, tremor, essencial tremor, ataxia, premutation

## Abstract

Fragile X-associated tremor/ataxia syndrome (FXTAS) is a cause of movement disorders and cognitive decline which has probably been underdiagnosed, especially if its prevalence proves similar to those of progressive supranuclear palsy and amyotrophic lateral sclerosis. We report a case of a 74-year-old man who presented with action tremor, gait ataxia and forgetfulness. There was a family history of tremor and dementia, and one of the patient’s grandsons was mentally deficient. Neuropsychological evaluation disclosed a frontal network syndrome. MRI showed hyperintensity of both middle cerebellar peduncles, a major diagnostic hallmark of FXTAS. Genetic testing revealed premutation of the *FMR1* gene with an expanded (CGG)_90_ repeat. The diagnosis of FXTAS is important for genetic counseling because the daughters of the affected individuals are at high risk of having offspring with fragile X syndrome. Tremors and cognitive decline should raise the diagnostic hypothesis of FXTAS, which MRI may subsequently reinforce, while the detection of the *FMR1* premutation can confirm the condition.

## Abstract (pt)

A síndrome de tremor-ataxia associada ao X-frágil (FXTAS) é uma causa de distúrbios do movimento e de declínio cognitivo que provavelmente tem sido subdiagnosticada, especialmente se a sua prevalência for realmente similar às da paralisia supranuclear progressiva e esclerose lateral amiotrófica. Relatamos um caso de um homem de 74 anos que se apresentou com tremor de ação, ataxia de marcha e esquecimento. Havia história familiar de tremor e de demência e um de seus netos era mentalmente deficiente. A avaliação neuropsicológica demonstrou uma síndrome frontal. A ressonância magnética (RM) revelou hiperintensidade de ambos os pedúnculos cerebelares médios, um critério maior para o diagnóstico de FXTAS. Os testes genéticos confirmaram a presença da pré-mutação do gene *FMR1*, com uma repetição (CGG)_90_. O diagnóstico de FXTAS é importante para o aconselhamento genético porque as filhas dos indivíduos afetados tem alto risco de ter uma criança com síndrome do X-frágil. A presença de tremores e declínio cognitivo deve levantar a hipótese diagnóstica de FXTAS, que poderá ser reforçada pela RM e confirmada pela presença da pré-mutação do gene *FMR1*.

The Fragile X-associated tremor/ataxia syndrome (FXTAS) is associated with premutation of the *FMR1* gene, located on the X chromosome.^1^ This premutation is characterized by the expansion of the CGG repeat at the 5’ untranslated region of the gene. While the repeats in the *FMR1* alleles in the general population contain six to 55 triplets, in premutations they vary from (CGG)_>55_ to (CGG)_200_. The premutations are functional and produce the protein (FMRP). However, they are unstable upon transmission to offspring and, when maternally transmitted, the premutation repeat may eventually expand to (CGG)_>200_, thus characterizing the full mutation that causes the Fragile X syndrome (FXS) as a result of FMRP deficiency.^2^ FXS is the most common cause of inherited mental retardation.^2,3^ FXTAS is not the only condition associated with the premutation of the *FMR1* gene. Premutated women tend to present ovarian insufficiency and about 20% of these cases have premature menopause.^4,5^

FXTAS typically manifests after the age of 50 with the late appearance of a tremor similar to essential tremor followed by ataxia, while cognitive decline, parkinsonism, peripheral neuropathy, proximal muscle weakness, and autonomic dysfunction have also been reported.^3^ The over-expression of the *FMR1* messenger RNA by the premutated alleles, which may be a consequence of a decrease in FMRP synthesis, has been considered a possible mechanism leading to FXTAS.^2^

In the first studies on the syndrome, FXTAS was considered to be exclusive to men, but shortly after, several cases of premutated women with a similar clinical picture were reported.^6-8^

The presence of executive cognitive deficits has been described since the initial publications on the subject,^2^ although more recent reports have described cases with dementia.^9,10^ The magnetic resonance imaging (MRI) features are rather typical,^11^ although not specific to FXTAS.^12,13^

Our aim was to describe the cognitive profile and neuroimaging features of a case of FXTAS and to highlight the importance of this diagnosis in the clinical practice.

## Case report

A 74 year-old man, a retired bank clerk, presented with hand tremors that had started five years earlier and worsened over the last year. The tremors were more severe when the patient had to hold objects with his hands or when he was worried. He also complained of gait disturbance attributed to previous traumatic lesions of the ankle and knee joints that occurred during his lifetime as a football (soccer) player. Recently, he had manifested several episodes of urinary incontinence and a few incidents of fecal incontinence. His wife reported that he had become progressively more forgetful over the last five years, with difficulties in remembering appointments, messages or where he had kept his things. In the last month he had presented episodes of misinterpretations of TV programs as real events and of not recognizing his house as his own. No other physical illness was reported. His mother had a similar tremor when she was very old. She died at the age of 94, but after 89 years she began to show cognitive decline. One of his sisters also has hand tremors but no memory complains, whereas his other sister and brother were apparently unaffected. He had two daughters and the only offspring of one of the daughters was a 7-year-old mentally deficient boy. His two sons were clinically normal.

On examination his blood pressure was 140 × 80 mm Hg, and there were signs of arthrosis in both ankle and knee joints. His gait was slightly ataxic and action (postural and kinetic) tremors in upper extremities, left greater than right, were evident. Rest tremor as well as bradykinesia and rigidity were absent. The hand tremors made it difficult to evaluate the coordination of the upper extremities, whereas no coordination problems were present in his legs. The subject also had voice tremor.

**Figure 1:**
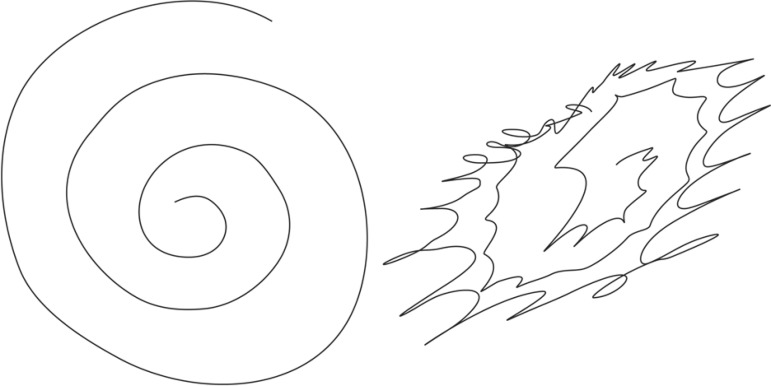
Action tremor seen when drawing the Archimedes’ spiral.

He scored 20 on the Mini-mental State Examination (MMSE),^14,15^ a low score given his 16 years of schooling, in spite of his incapacity for drawing or writing because of the tremor (Figure 1). He was easily distracted and was unable to accurately repeat sentences from the MMSE or to obey the simple commands involved in the test. In the memorization of drawings from the Brief Cognitive Battery, he showed more severe learning than delayed recall impairment demonstrated by his score of 5 out of the 10 items in the learning test, while in the delayed recall test he was able to recall 4 of the 5 items.^16,17^ In the Dementia Rating Scale he scored 88, with low performance across all subtests (drawing and writing incapacities further reduced his score).^18,19^ He scored 3 on the Digit span in both direct and reverse order. In the Logical Memory of the Wechsler Memory Scale he attained the 6^th^ percentile on immediate recall, but was able to reach the 10^th^ percentile after 30 minutes.^19^ Semantic verbal fluency was low for animals in one minute (5 animals) as well as for supermarket items (6 items). Phonemic verbal fluency was also very low, with a total score of 4 for the letters F, A and S together, and 2 for the letter P. His score on the Stroop Color Test was poor with 22 errors (1.8 standard deviations below the mean for his age).^21,22^ His score on the Questionnaire of Functional Activities was 7 (scores higher than 5 are indicative of impairment in instrumental activities of daily living, often observed in dementias).^23^

**Figure 2:**
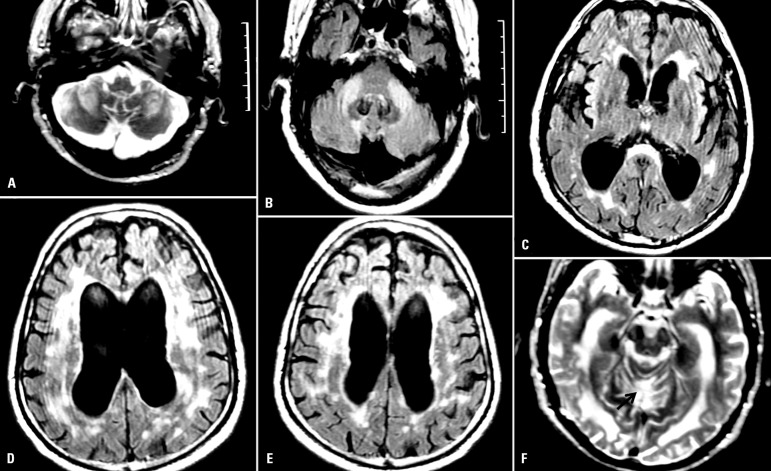
Hyperintensities in both middle cerebellar peduncles seen in T2 (A) and in FLAIR acquisitions(B); ventricle dilatation, hyperintensities in periventricular white matter and in splenium of corpus callosum in FLAIR(C); hyperintensities in the periventricular and brain white matter, ventricle dilatation and brain atrophy in FLAIR (D,E); arrow pointing to a possible vermian infarction in FLAIR (F).

Routine laboratory tests were normal except for a high serum level of triglycerides. EKG was normal. Brain MRI images were acquired on a 1.5T MR system (Signa Excite - GE - Milwaukee - USA) using an 8 Ch head coil. Axial T2-weighted FSE (TR: 2200 ms / TEef: 90 ms / NEX: 1), axial FLAIR (TR: 11000 ms / TEef: 104 ms / TI: 2200 ms / NEX: 1); pre and post Gadolinium axial MTC T1-weighted (TR: 500 ms / TE: 19 ms / TI: ms / NEX: 1 / MT offset: 1200 Hz) images were collected as per routine investigation. There was marked ventricle enlargement, with slight augmentation of the remaining CSF spaces. Diffuse T2 and FLAIR white matter hyperintensities were found in both superior and infratentorial compartments, with a distinct distribution, being symmetrical in the middle cerebellar peduncles (MCP), splenium of the corpus callosum, middle pontine region and periaqueductal grey matter - and most of the bi-hemispheric white matter and periventricular regions (Figure 2). These lesions did not exhibit contrast enhancement and had no corresponding MR signal alterations on the Magnetization Transfer Contrast images. Additionally, an area of tissue loss located in the anterior vermis (at the culmen and declive lobules) was detected indicating a possible earlier infarct.

Analysis of the FMR1 gene showed a premutation with (CGG)_90_. The presence of the premutation in this patient prompted the genetic counseling of his two daughters, obligate permutation carriers. The examination of his mentally retarded grandson revealed the *FMR1* full mutation, thus establishing the diagnosis of fragile X syndrome.

He was treated with primidone 100 mg/day associated with propranolol 40 mg/day with partial improvement in the tremor, making eating and shaving easier for him.

An informed consent form was signed by the patient’s son authorizing the publication of the case for medical and scientific purposes.

## Discussion

According to criteria proposed by Jacquemont et al. this was a definite case of FXTAS because the major criteria consisting of tremor, ataxia and the MRI findings of T2 and FLAIR hyperintensities in the middle cerebellar peduncles were fulfilled in a patient with the *FMR1* gene premutation.^24^ Other key features pointing to the diagnostic hypothesis were the presence of cognitive decline and mental retardation in a grandson. Hyperintensities in the deep white matter and brain atrophy present in this case are also frequent in FXTAS.^2,11^ Additionally, the patient also presented a sign of a possible earlier infarct in the cerebellar vermis.

The action tremor was the main concern of this patient, but for his wife the cognitive decline was the foremost reason to seek medical advice. The complaint of forgetfulness in a man in his seventies usually raises the possibility of the diagnosis of Alzheimer’s disease (AD). However, even his performance on the MMSE was atypical for AD, because he was unable to obey the simple commands or to repeat short sentences. These two sub-items of the MMSE, which are related to attention and working memory, are usually performed easily by patients with mild or moderate dementia in AD. The neuropsychological evaluation disclosed that delayed recall was less affected than immediate memory and revealed very low semantic and phonemic verbal fluencies as well as many errors on the Stroop test. This cognitive profile or pattern is more reminiscent of the frontal or frontal-subcortical type of dementia or progressive frontal network syndrome,^25^ and has been described in FXTAS.^9,26^ In a comprehensive neuropsychological evaluation of an FXTAS case, it was reported that control of attention, working memory, executive functioning, and both declarative and procedural learning were the main impaired functions whereas speech and language were essentially normal, where visual and spatial abilities were relatively unimpaired, and verbal reasoning was only slightly compromised.^26^ This pattern may be also seen in vascular dementia, a diagnosis that could be reinforced by the diffuse white matter changes and by the possible cerebellar infarct seen in this case. Although the diffuse white matter change has been described in FXTAS^2^ and the action tremor is probably not related to this possible cerebellar vermis infarct, cerebrovascular disease may be an associated condition in this case.

In FXTAS there are ubiquitin-positive intranuclear inclusions in neurons and astrocytes diffusely in the brain. The inclusions are spherical, contain granulofilamentous material and predominate in the hippocampus. In the white matter there are patches of pallor and spongiosis with myelin and axonal losses, which correspond to the areas of T2 hyperintensity on MRI.^2^

FXTAS can occur in approximately 40% of male premutation carriers older than 50 years, and the lifetime risk for the general male population is around 1 in 3000 to 6000, which makes the expected prevalence of FXTAS similar to those of inherited ataxia, progressive supranuclear palsy, multiple system atrophy, and amyotrophic lateral sclerosis.^2^ FXTAS is usually less severe in women and dementia is most likely rare.^7^

The most important differential diagnosis of FXTAS is essential tremor because action tremor is an initial manifestation of FXTAS and essential tremor is very frequent in the general population. It should be noted that essential tremor usually manifests at an earlier age, between 35 and 45 years,^27^ while the tremor of FXTAS manifests around the age of 60 years.^2^ When ataxia is also present, inherited ataxias, multiple system atrophy of cerebellar type and vascular disease may be included in the possible diagnosis.^2,13^, although it should be noted that dementia is not a manifestation of multiple system atrophy. In these conditions, MRI findings may also be similar to those described in FXTAS.^11-13^ MRI changes of FXTAS usually precede clinical manifestations.^28,29^

There is no specific treatment for FXTAS that targets the underlying pathogenic mechanism of excess FMR1 mRNA. However, there are several approaches for treating specific neurological and psychiatric signs as well as supportive intervention.^30^

The action tremor may show partial improvement with primidone or propranolol as observed in the present case. Cholinesterase inhibitors have been tested in cognitive impairment with controversial results.^2^

In spite of the lack of effective treatment it is important to diagnose FXTAS for genetic counseling because daughters of the patients inherit the premutation and are at high risk of having offspring with fragile X syndrome. Clinicians should test for FMR1 premutation if the patient has late onset-action tremor, cerebellar ataxia of unknown cause, dementia associated with movement disorders, prior diagnosis of multiple system atrophy of the cerebellar subtype and MCP sign on T2/FLAIR MRI. FXTAS should also be suspected when there is a familial history of FXS, relatives with mental retardation and no clear diagnosis, family or patient history of infertility or premature menopause associated with tremors or other signs suggestive of FXTAS.

## References

[1] Hagerman RJ, Leehey M, Heinrichs W (2001). Intention tremor, parkinsonism, and generalized brain atrophy in
male carriers of fragile X. Neurology.

[2] Amiri K, Hagerman RJ, Hagerman PJ (2008). Fragile X-associated tremor/ataxia syndrome: an aging face of the
fragile X gene. Arch Neurol.

[3] Leehey MA, Munhoz RP, Lang AE (2003). The fragile X premutation presenting as essential
tremor. Arch Neurol.

[4] Allingham-Hawkins DJ, Babul-Hirji R, Chitayat D (1999). Fragile X premutation is a significant risk factor for premature
ovarian failure: the International Collaborative POF in Fragile X
study-preliminary data. Am J Med Genet.

[5] Vianna-Morgante AM, Costa SS, Pavanello RCM, Otto PA, Regina C, Mingroni-Netto RC (1999). Premature ovarian failure (POF) in Brazilian fragile X
carriers. Genet Mol Biol.

[6] Horvath J, Burkhard PR, Morris M, Bottani A, Moix I, Delavelle J (2007). Expanding the phenotype of fragile X-associated tremor/ataxia
syndrome: a new female case. Mov Dis.

[7] Hagerman RJ, Leavitt BR, Farzin F (2004). Fragile-X-associated tremor/ataxia syndrome (FXTAS) in females
with the FMR1 premutation. Am J Hum Genet.

[8] O'Dwyer JP, Clabby C, Crown J, Barton DE, Hutchinson M (2005). Fragile X-associated tremor/ataxia syndrome presenting in a woman
after chemotherapy. Neurology.

[9] Gonçalves MRR, Capelli LP, Nitrini R (2007). Atypical clinical course of FXTAS: rapidly progressive dementia
as the major symptom. Neurology.

[10] Greco CM, Hagerman RJ, Tassone F (2002). Neuronal intranuclear inclusions in a new cerebellar
tremor/ataxia syndrome among fragile X carriers. Brain.

[11] Brunberg JA, Jacquemont S, Hagerman RJ (2002). Fragile X premutation carriers: characteristic MR imaging
findings of adult male patients with progressive cerebellar and cognitive
dysfunction. Am J Neuroradiol.

[12] Okamoto K, Tokiguchi S, Furusawa T (2003). MR features of diseases involving bilateral middle cerebellar
peduncles. Am J Neuroradiol.

[13] Storey E, Billimoria P (2005). Increased T2 signal in the middle cerebellar peduncles on MRI is
not specific for fragile X premutation syndrome. J Clin Neurosci.

[14] Folstein MF, Folstein SE, McHugh PR (1975). "Mini-mental State": a practical method for grading the cognitive
state of patients for the clinician. J Psychiatr Res.

[15] Brucki SMD, Nitrini R, Caramelli P, Bertolucci PHF, Okamoto IH (2003). Sugestões para o uso do Mini-Exame do Estado Mental no
Brasil. Arq Neuropsiquiatr.

[16] Mattis S (1988). Dementia Rating Scale: professional manual.

[17] Porto SC, Charchat-Fichman H, Caramelli P, Bahia VA, Nitrini R (2003). Dementia Rating Scale - DRS - in the diagnosis of patients with
Alzheimer's dementia. Arq Neuropsiquiatr.

[18] Nitrini R, Lefèvre BH, Mathias SC (1994). Neuropsychological tests of simple application for diagnosing
dementia. Arq Neuropsiquiatr.

[19] Nitrini R, Caramelli P, Herrera E Jr (2004). Performance of illiterate and literate nondemented elderly
subjects in two tests of long-term memory. J Int Neuropsychol Soc.

[20] Weschler D (1987). Weschler Memory Scale: revised manual.

[21] Stroop JR (1935). Studies of interference in serial verbal
reactions. J Exp Psychol.

[22] Duncan MT (2006). Assessment of normative data of Stroop test performance in a
group of elementary school students in Niterói. J Bras Psiquiatr.

[23] Pfeffer RI, Kurosaki TT, Harrah CH Jr, Chance JM, Filos S (1982). Measurement of functional activities in older adults in the
community. J Gerontol.

[24] Jacquemont S, Hagerman RJ, Leehey M (2003). Fragile X premutation tremor/ataxia syndrome: molecular,
clinical, and neuroimaging correlates. Am J Hum Genet.

[25] Mesulam MM (2000). Principles of behavior and cognitive neurology.

[26] Grigsby J, Leehey MA, Jacquemont S (2006). Cognitive impairment in a 65-year-old male with the fragile
X-associated tremor-ataxia syndrome (FXTAS). Cog Behav Neurol.

[27] Lou JS, Jankovic J (1991). Essential tremor: clinical correlates in 350
patients. Neurology.

[28] Loesch DZ, Litewka L, Churchyard A, Gould E, Tassone F, Cook M (2007). Tremor/ataxia syndrome and fragile X premutation: diagnostic
caveats. J Clin Neurosci.

[29] Capelli LP, Goncalves MRR, Kok F (2007). Fragile X-associated tremor/ataxia syndrome: intrafamilial
variability and the size of the FMR1 premutation CGG repeat. Mov Dis.

[30] Berry-Kravis E, Abrams L, Coffey SM (2007). Fragile X-associated tremor/ataxia syndrome: clinical features,
genetics, and testing guidelines. Mov Disord.

